# 

**Published:** 1878-07-20

**Authors:** 


### ORIGINAL AND SELECTED ARTICLES.

Tubercular Meningitis, treated with Iodia; by J. W. Unger, M.D., of Miss....193
Clinical Reports of the Demilt Dispensary; by Dr. L. Duncan Bulkley....194
The Use of Sulphate of Cinchonidia in Acute Bright's Disease and in Intermittent Fever; by J. C. McMechan, M.D....198
Congenital Phimosis with Adherent Prepuce; by E. C. Lemen, M.D....199
The Treatment of Sore Nipples....201
A Bloodless Method of Performing Tracheotomy....202
Arsenic....203
Intravenous Injection of Milk....303

### ABSTRACTS AND GLEANINGS.

The Metric System in a Nut Shell, 206; Diseases of the Eye, 208; Arm Presentations, 209; Urethral Stricture, 210; Injuries of the Head, 211; The Medical Treatment of Children, 213; Varicocele Treated by Subcutaneous Ligation, 214; Treatment of Pneumonia, 214; Prophylactic Treatment of Placenta Prævia, 215; Mate or Paraguay Tea, 216; The Microphone in Medicine, 216; New Plan of Tying Knots in Surgical Operations, 216; Bromide of Arsenic in the Treatment of Nervous Diseases, 217; Daturia as a Mydriatic, 217; Action of Strychnia on the Eye, 217; Muriate of Ammonia for the Hydrocele of Infants, 217; Post Partum Hemorrhage, 217.

### PRACTICAL NOTES AND FORMULÆ.

How shall Quinia be Dispensed, 218; Remarks on Officinal Syrups, 218; McCall's Anti Dyspeptic Powders, 218; Apthous Ulcerations, 219; Strychnia and Dogs, 219; Hydrargyrum Cum Creta, 219; Simple Elixir, 219; Nervousness and Tobacco, 219; Arsenic in Bismuth, 219; Wine of Gentian, 220; Anti-Colic Mixture, 220; Santonine Poisoning, 220; Asthma, 220; Whooping Cough, 220; Tincture Stoppers, 220; Diagnosis of Pregnancy, 220; Spirits of Chloroform, 220; Camphenyl, 220; Dysmenorrhœa, 220; For Constipation, 220.

### SCIENTIFIC ITEMS.

The Telephone in Ausculation, 221; A Singular Fire, 221; Sea Cables and Fish, 221; The Microphone, 221.

### EDITORIAL AND MISCELLANEOUS.

The Walking Man, 222; Dissecting Materiel, 222; Fucus Vesiculosis, 223; Book Notices, 223; Death of Dr. E. M. Campbell, of Virginia, 224; Hygeian Home, 224; Notices of New Advertisements, 224.

